# Adolescent Cardiorespiratory Fitness and Future Work Ability

**DOI:** 10.1001/jamanetworkopen.2024.3861

**Published:** 2024-03-27

**Authors:** Perttu T. T. Laakso, Francisco B. Ortega, Pertti Huotari, Asko J. Tolvanen, Urho M. Kujala, Timo T. Jaakkola

**Affiliations:** 1 Faculty of Sport and Health Sciences, University of Jyväskylä, Jyväskylä, Finland; 2 Department of Physical Education and Sports, Faculty of Sport Sciences, Sport and Health University Research Institute (iMUDS), University of Granada and CIBERobn Physiopathology of Obesity and Nutrition, Granada, Spain; 3 Faculty of Education and Psychology, University of Jyväskylä, Jyväskylä, Finland

## Abstract

**Importance:**

Although research indicates that low fitness in youth is associated with a higher risk of chronic disability in men, the association of fitness in adolescence with work ability in working men and women remains unknown.

**Objective:**

To examine the associations of adolescent health-related physical fitness with future work ability.

**Design, Setting, and Participants:**

This 45-year observational cohort study, conducted in Finland, examined the direct and indirect longitudinal associations of objectively measured health-related fitness in adolescence (in 1976) with self-reported work ability and sickness absence in early middle age (in 2001) and the Work Ability Index in late middle age (in 2021). A countrywide stratified random baseline sample included fitness measurements for cardiorespiratory fitness (CRF) (running 1.5 km for girls and 2 km for boys), musculoskeletal fitness (MF; standing broad jump and sit-ups for both sexes, pull-ups for boys, and flexed-arm hang for girls), and height and weight, from which body mass index (BMI) was calculated. Structural equation modeling–based path analysis, adjusted for age and sex at baseline and for educational level, work-related physical strain, and leisure-time physical activity in late middle age, was conducted. Data analysis was performed from January to July 2023.

**Main Outcomes and Measures:**

Self-reported work ability was measured with structured questions in early middle age and with the validated Work Ability Index in late middle age.

**Results:**

The final sample from longitudinal analyses (1207 individuals; 579 [48%] male individuals) consisted of participants with fitness measurement from age 12 to 19 years, and work ability assessment from age 37 to 44 years and/or age 57 to 64 years. Higher adolescent CRF was associated with higher work ability (839 participants; β = 0.12; 95% CI, 0.01 to 0.22; *P* = .03) and lower sickness absence (834 participants; β = −0.07; 95% CI, −0.12 to −0.02; *P* = .004) in early middle age and, indirectly, mediated by work ability in early middle age, with a higher work ability at the end of working age (603 participants; β = 0.04; 95% CI, 0.001 to 0.08; *P* = .04). The results remained consistent in both sexes and after adjustment for the confounders. Neither MF nor BMI was associated with work ability (MF, 1192 participants; β = −0.07; 95% CI, −0.17 to 0.03; BMI, 1207 participants, β = 0.09; 95% CI, −0.004 to 0.19) or sickness absence (MF, 1185 participants, β = 0.02; 95% CI, −0.03 to 0.06; BMI, 1202 participants, β = −0.03; 95% CI, −0.09 to 0.03) in early middle age or with late middle age work ability, mediated by work ability in early middle age (MF, 603 participants, β = −0.02; 95% CI, −0.06 to 0.01; BMI, 603 participants, β = 0.03; 95% CI, −0.004 to 0.07).

**Conclusions and Relevance:**

These findings suggest that low CRF in youth is associated with poor work ability at the middle and end of working life, which highlights the informative and prognostic value of CRF assessment early in youth. Enhancing CRF in the first decades of life might contribute to better work capacity and productivity in the labor force, which would have implications for health, quality of life, society, and the economy.

## Key Points

**Question:**

Is health-related physical fitness during adolescence associated with work ability at the middle and end of working life?

**Findings:**

In this cohort study of 1207 individuals in Finland, higher cardiorespiratory fitness, but not musculoskeletal fitness or body mass index, in adolescence was associated with higher work ability and lower sickness absence in adulthood.

**Meaning:**

These findings suggest that low cardiorespiratory fitness in youth may be an early indicator of impaired work ability throughout working life, and enhancing cardiorespiratory fitness in the first decades of life might contribute to better work capacity and productivity in the labor force.

## Teaser

This cohort study examines the associations of adolescent health-related physical fitness with future work ability among individuals in Finland.

## Introduction

Work ability refers to individuals’ work-related physical, mental, and psychosocial resources and factors associated with the working environment.^1^ Low educational level, older age, a high physical and mental workload, and impaired health status have been identified as negatively affecting work ability.^2,3,4^ Maintaining good work ability is essential at all societal levels. For the individual, it increases well-being, quality of life, and employment,^2,5,6^ and for the work organization it prevents disability,^7,8^ sickness absence,^8,9^ and presenteeism^10^ (ie, working while ill^11^), which have been associated with substantial costs.^12,13^ At the community level, work ability is positively associated with gross domestic product.^14,15^

Health-related physical fitness (hereafter fitness) is a dimension of physical fitness defined as the ability to perform physically demanding tasks acceptably. The components of fitness are cardiorespiratory fitness (CRF), flexibility, body composition, and musculoskeletal fitness (MF).^16^ CRF, MF, and healthy body composition are positively associated with both current^17,18,19,20^ and future^21,22,23,24,25^ health. Assessing fitness in childhood and adolescence is important, because it has been shown to be an important marker of noncommunicable diseases.^22,25,26^

The association of fitness with work ability has been demonstrated in cross-sectional studies. Evidence from experimental^27,28^ and observational^4,29^ studies has shown that sufficient levels of CRF^27,28,29^ and MF^4,30^ and a healthy body weight^3,4,31^ benefit work ability. However, studies using objective fitness measurement have relied on small samples, which limits their generalizability. In turn, longitudinal studies on the benefits of youth fitness in association with work ability have only investigated the association for adulthood chronic disability and mostly in men.^32,33,34,35^ It is, therefore, unknown whether lower youth fitness levels are associated with lower work ability in persons in the labor force (ie, those not receiving a disability pension) and the possible economic consequences of this. The corresponding associations in women also remain unknown.

Public debate has raised concern on whether deteriorating fitness^36^ in youth will lead to a future labor force with greatly eroded work ability. At the same time, the working-age population in high-income countries is decreasing,^37^ prompting calls to maximize the output of the existing labor force. This cohort study investigated the associations of adolescent fitness with work ability in early and late middle age among male and female participants. Specifically, we aimed to provide novel evidence on the associations of fitness in adolescence with future work ability in both sexes and ascertain the informative value of fitness based on a uniquely long follow-up study.

## Methods

### Study Population

**Figure 1.**
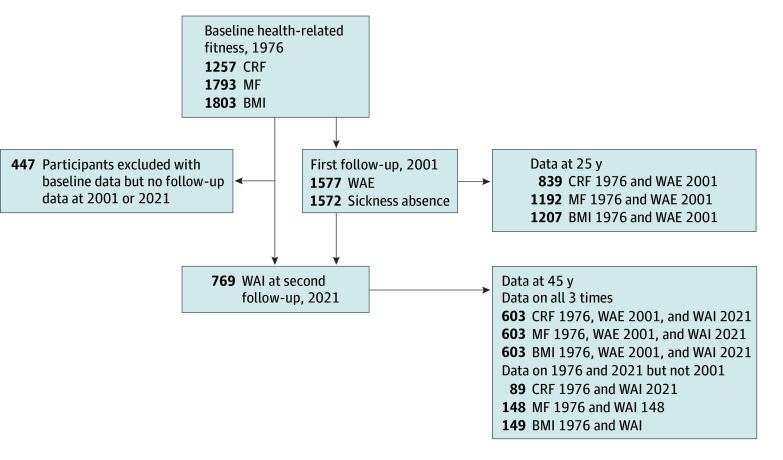
Data Structure of the Study The path model used in this study analyzed all the associations of at least 1 exposure in baseline (1976) with at least 1 outcome in the first (2001) and/or the second (2021) follow-up. Because of the possibility of participating in 1976 and 2021 without participating in 2001, the missing data are not cumulative. The numbers for a particular association analyzed are presented in eTable 1 in Supplement 1. BMI indicates body mass index; CRF, cardiorespiratory fitness; MF, musculoskeletal fitness; WAE, Work Ability Estimate; WAI, Work Ability Index.

This cohort study used data collected at 3 time points for the Liikuntaharrastuksen Seurantatutkimus (ie, Physical Activity Follow-Up Study) project, which was launched in 1976: (1) baseline was in April to May 1976, (2) follow-up 1 was in April to May 2001, and (3) follow-up 2 was in March 2021. The baseline sample comprised children and adolescents aged 9 to 21 years, of whom 12- to 19-year-old adolescents (mean [SD] age, 14.9 [1.7] years) with eligible fitness data were qualified for inclusion in the final sample (up to 1803 participants). The baseline sample was recruited using stratified random sampling, from 56 schools in the eastern, western, central, and northern parts of Finland, including urban and rural districts. The follow-up 1 data, which were collected with a postal questionnaire when the participants were aged 37 to 44 years, provided information on early middle age work ability (up to 1577 participants, 87.5% of baseline sample). The follow-up 2 data, comprising 769 participants (43% of baseline sample), then aged 57 to 64 years, provided information on late middle age Work Ability Index (WAI) data. The Liikuntaharrastuksen Seurantatutkimus data acquisition method has been described in detail elsewhere.^38^ Because of participant dropout and missing data in the baseline fitness test results and follow-up item responses, the number of participants varied depending on the longitudinal association examined. The exact number of participants in each analysis is presented in eTable 1 in Supplement 1, and the data structure is shown in Figure 1. The Human Sciences Ethics Committee of the University of Jyväskylä approved the study. Written informed consent was obtained before study entry. The study follows the Strengthening the Reporting of Observational Studies in Epidemiology (STROBE) reporting guidelines.

### Assessment of Physical Fitness

The baseline physical fitness tests were administered in schools by a trained research team using an identical measurement protocol. The tests used were based on the International Fitness Test guidelines.^39^ Before tests, participants completed a short health inquiry,^40^ and only healthy adolescents were allowed to participate. Participants conducted an identical warm-up routine, and their height (centimeters) and weight (kilograms) were measured by their school health care professional. CRF was assessed by running tests (2000 m for boys and 1500 m for girls) performed on an outdoor 400-m running track. MF tests, conducted in an indoor sport facility, included standing broad jump (centimeters), sit-ups (repetitions in 30 seconds), and pull-ups (maximum number of repetitions) and/or flexed-arm hang (seconds). The standing broad jump and sit-up tests were identical for both sexes, whereas the upper-body strength tests were sex specific, with pull-ups for boys and flexed-arm hang for girls. The tests are given in detail in eTable 2 in Supplement 1. For statistical analyses, the test scores were adjusted for age and sex by calculating standardized *z* scores.

### Assessment of Work Ability

In late middle age (2021), work ability was self-assessed using the WAI^41^ (eTable 3 in Supplement 1). Participants who were retired (17 participants), disabled (2 participants), or otherwise permanently outside working life (12 participants) were excluded. The total WAI score ranges between 7 and 49. Scores can subsequently be categorized according to the original classification^4^ as poor (7-27), average (28-36), good (37-43), and very good (44-49).

Because the WAI was not used in the follow-up 1 questionnaire in 2001, we used the Work Ability Estimate^2,42,43^ and sickness absence as compensatory indicators of work ability. The Work Ability Estimate (hereafter, work ability) is a single-question self-assessment method used previously in the Finnish population-based Mini-Finland study.^42,43^ The question is formulated as, “Do you have any disease or injury which decreases your work ability and overall functioning (no/yes)?” The variable was subsequently inverted by multiplying it by −1 to align it with the WAI so that for both outcomes the higher the score represented the higher the level of work ability. Sickness absence, which has shown a negative reciprocal association with work ability,^7,8,9,10^ was assessed as the number of full days off work over the past 12 months. Both indicators are included in the WAI.^41^

### Covariates

At baseline (1976), the fitness test results were adjusted for age and sex by calculating standardized *z* scores. Educational level, work-related physical strain, and leisure-time physical activity (LTPA) in late middle age (2021) were added into the path model to act as cross-sectional variables and as covariates in the longitudinal associations. Formal educational level was obtained by asking, “How many years in total have you studied full-time, including all levels of education?” Work-related physical strain by asking the structured question, “What kind of work do you do most of the year (information, information and physical, or physical work)?” LTPA was assessed as frequency (“How often do you participate in physical activity?”), duration (“What is the duration of your average LTPA session?”), and intensity (“How would you describe the intensity of your average LTPA session?”),^35^ from which metabolic equivalent of task (MET) hours per day was calculated.

### Statistical Analysis

Data analysis was performed from January to July 2023. Longitudinal associations between the adolescent fitness components, early middle age work ability indicators, and the late middle age WAI were investigated using structural equation modeling–based path analysis, conducted with Mplus version 8.0 (Muthen & Muthen). On the basis of the results of the sensitivity analyses described in the eAppendix in Supplement 1, a single group model combining sex and age groups was adopted as a final model. The mediation effect was tested via the indirect effect estimate using a robust SE estimate.

Model fit was evaluated using the χ^2^ test, root mean square error of approximation, comparative fit index, Tucker-Lewis index, and standardized root mean square residuals. Models were estimated using the weighted least squares mean and variance estimator with robust SE and scale-corrected χ^2^ value. Missing data analysis was conducted with the Little missing completely at random test using SPSS statistical software version 28 (IBM). All *P* values were 2-sided, and the significance threshold was set at *P* < .05.

## Results

### Descriptive Statistics

Table 1 shows the participants’ baseline characteristics, and Table 2 shows the follow-up characteristics. The study was well-balanced by sex (1207 individuals; 579 male [48%]). The prevalence of decreased work ability in early middle age was 18.0% (134 participants) in men and 16.8% (140 participants) in women, and the mean (SD) sickness absence rate was 10.6 (44.2) full days per year among men and 9.4 (35.1) full days per year among women. In late middle age, 56.7% of the participants (436 participants) worked in low and 14.1% (108 participants) worked in high physically demanding jobs. The mean (SD) WAI value (39.1 [7.0] for men; 37.9 [7.3] for women) was defined as good, according to the original categorization. A significant decrease in the WAI by age was observed among women (mean [SD], 38.6 [7.3] for age 57-60 years vs 36.4 [7.2] for age 61-64 years; *t*_415_ = 8.18; *P* = .004) but not among men (mean [SD], 39.1 [7.0] for age 57-60 years vs 39.2 [7.2] for age 61-64 years; *t*_374_ = 0.00; *P* = .95). The mean (SD) LTPA value was 3.5 (3.4) MET hours per day among men and 2.8 (2.5) MET hours per day among women, and those with higher LTPA tended to have higher WAI. One-way analysis of variance with categorized LTPA variable (high LTPA, >3.50 MET hours per day; middle LTPA, >1.57 to ≤3.50 MET hours per day; low LTPA, ≤1.57 MET hours per day) showed that the groups of high (mean [SD] WAI, 40.1 [6.6]) and middle (mean [SD], 39.3 [6.7]) LTPA had a significantly higher mean WAI (analysis of variance *F*_2,754_ = 9.59; *P* < .001 for high vs low; *P* = .02 for middle vs low) than the low LTPA group (mean [SD], 37.6 [7.1]) (Scheffe pairwise comparison). Correlations between the study variables are presented in Table 3.

**Table 1.** Baseline Characteristics of the Study Sample

Characteristic	Male participants						Female participants					
	12-19 y		12-15 y		16-19 y		12-19 y		12-15 y		16-19 y	
	Mean (SD)	No.^a^	Mean (SD)	No.^a^	Mean (SD)	No.^a^	Mean (SD)	No.^a^	Mean (SD)	No.^a^	Mean (SD)	No.^a^
Age, y	14.9 (1.7)	NA	13.8 (1.0)	NA	16.8 (0.9)	NA	14.9 (1.7)	NA	13.9 (1.0)	NA	16.8 (0.9)	NA
Height, cm	168.2 (11.2)	872	163.4 (10.6)	562	176.6 (6.4)	310	162.0 (6.5)	931	160.7 (6.4)	615	164.6 (5.8)	316
Weight, kg	56.2 (12.4)	872	51.1 (10.9)	562	65.2 (9.4)	310	52.5 (8.2)	931	50.7 (8.5)	615	56.1 (6.8)	316
Body mass index^b^	19.6 (2.7)	872	18.9 (2.5)	562	20.9 (2.6)	310	20.0 (2.5)	931	19.6 (2.6)	615	20.7 (2.2)	316
Sit-ups in 30 s	20.4 (4.2)	866	19.6 (4.2)	551	21.7 (3.8)	315	16.6 (3.8)	934	16.5 (3.7)	611	16.7 (4.0)	323
Standing broad jump, cm	213.2 (30.1)	868	200.5 (25.4)	551	235.5 (24.0)	317	174.1 (21.4)	940	171.9 (21.2)	613	178.4 (21.2)	327
Flexed arm hang, s	NA	NA	NA	NA	NA	NA	13.9 (9.9)	939	13.5 (10.0)	612	14.6 (9.7)	327
Pull-ups	5.7 (4.0)	862	4.6 (3.5)	548	7.6 (4.2)	314	NA	NA	NA	NA	NA	NA
Running test												
1500 m, s	NA	NA	NA	NA	NA	NA	495.6 (87.0)	587	501.0 (87.2)	422	480.0 (85.0)	165
2000 m, s	582.5 (121.1)	660	601.4 (124.1)	438	545.7 (106.2)	222	NA	NA	NA	NA	NA	NA

Abbreviation: NA, not applicable.

^a^ Refers to number of participants.

^b^ Body mass index is calculated as weight in kilograms divided by height in meters squared.

**Table 2.** Follow-Up Characteristics of the Study Sample

Characteristic	Male participants												Female participants											
	25-y Follow-up						45-y Follow-up						25-y Follow-up						45-y Follow-up					
	37-44 y		37–40 y		41-44 y		57-64		57-60 y		61-64 y		37-44 y		37-40 y		41-44 y		57-64 y		57-60 y		61-64 y	
	Mean (SD)	No.^a^	Mean (SD)	No.^a^	Mean (SD)	No. ^a^	Mean (SD)	No.^a^	Mean (SD)	No.^a^	Mean (SD)	No.^a^	Mean (SD)	No.^a^	Mean (SD)	No.^a^	Mean (SD)	No.^a^	Mean (SD)	No.^a^	Mean (SD)	No.^a^	Mean (SD)	No.^a^
Age, y	39.9 (1.7)	NA	38.3 (1.0)	NA	41.8 (0.9)	NA	59.9 (1.7)	NA	58.3 (1.0)	NA	61.8 (0.9)	NA	39.9 (1.7)	NA	38.9 (1.0)	NA	41.8 (0.9)	NA	59.9 (1.7)	NA	58.9 (1.0)	NA	61.8 (0.9)	NA
Height, cm	179.6 (6.4)	739	179.6 (6.5)	471	179.7 (6.2)	268	176.7 (8.8)	366	176.5 (9.0)	234	177.1 (8.5)	132	165.9 (5.7)	814	165.9 (5.8)	531	165.8 (5.4)	283	167.7 (7.1)	403	167.8 (7.2)	269	167.6 (7.1)	134
Weight, kg	83.1 (12.0)	739	83.3 (12.2)	471	83.0 (11.8)	268	83.5 (14.5)	366	83.7 (15.0)	234	83.1 (13.4)	132	66.4 (11.6)	814	66.7 (12.4)	531	65.8 (10.0)	283	76.2 (16.5)	403	76.7 (17.4)	269	75.1 (14.5)	134
BMI^b^	25.7 (3.3)	739	25.8 (3.4)	471	25.7 (3.2)	268	26.7 (3.9)	366	26.8 (4.2)	234	26.4 (3.5)	132	24.1 (3.9)	814	24.2 (4.1)	531	24.0 (3.7)	283	27.0 (5.3)	403	27.2 (5.6)	269	26.7 (4.9)	134
Decreased work ability, No. (%)	134 (18.0)	745	82 (17.3)	473	52 (19.1)	272	NA	NA	NA	NA	NA	NA	140 (16.8)	832	99 (18.2)	544	41 (14.2)	288	NA	NA	NA	NA	NA	NA
Sickness absence, d/y	10.6 (44.2)	740	10.9 (44.2)	471	10.1 (44.2)	269	NA	NA	NA	NA	NA	NA	9.4 (35.1)	832	9.1 (33.9)	546	9.8 (37.4)	286	NA	NA	NA	NA	NA	NA
WAI	NA	NA	NA	NA	NA	NA	39.1 (7.0)	365	39.1 (7.0)	237	39.2 (7.2)	128	NA	NA	NA	NA	NA	NA	37.9 (7.3)	404	38.6 (7.3)	271	36.4 (7.2)	133
LTPA, MET-h/d	NA	NA	NA	NA	NA	NA	3.5 (3.4)	358	3.4 (3.4)	229	3.7 (3.3)	129	NA	NA	NA	NA	NA	NA	2.8 (2.5)	399	2.9 (2.7)	265	2.6 (2.1)	134
Strain, No. (%)																								
Low	NA	NA	NA	NA	NA	NA	210 (58.2)	361	130 (56.6)	231	80 (61.2)	130	NA	NA	NA	NA	NA	NA	226 (55.4)	408	157 (57.3)	273	69 (51.4)	135
Middle	NA	NA	NA	NA	NA	NA	88 (24.5)		57 (24.4)		31 (24.6)		NA	NA	NA	NA	NA	NA	136 (33.3)		77 (28.3)		59 (43.5)	
High	NA	NA	NA	NA	NA	NA	63 (17.3)		44 (19.0)		19 (14.2)		NA	NA	NA	NA	NA	NA	46 (11.3)		39 (14.3)		7 (5.1)	
Education, No. (%)																								
Basic (0-9 y)	NA	NA	NA	NA	NA	NA	13 (3.5)	365	9 (3.7)	247	4 (3.0)	118	NA	NA	NA	NA	NA	NA	5 (1.2)	404	3 (1.1)	273	2 (1.4)	131
Secondary (10-12 y)	NA	NA	NA	NA	NA	NA	69 (18.9)		49 (19.8)		20 (17.2)		NA	NA	NA	NA	NA	NA	64 (15.9)		56 (20.5)		8 (6.5)	
Higher (≥13 y)	NA	NA	NA	NA	NA	NA	283 (77.7)		189 (76.4)		94 (79.9)		NA	NA	NA	NA	NA	NA	335 (82.9)		214 (78.4)		121 (92.0)	

Abbreviations: BMI, body mass index; LTPA, leisure-time physical activity; MET-h/d, metabolic equivalent of task hours per day; NA, not applicable; WAI, Work Ability Index.

^a^ Refers to number of participants.

^b^ Body mass index is calculated as weight in kilograms divided by height in meters squared.

**Table 3.** Correlations Between the Study Variables^a^

Year and variable	1976			2001		2021		
	BMI	MF	CRF	Work ability^b^	Absence^c^	WAI	LTPA	Strain^d^
1976								
MF	−0.226^e^	NA	NA	NA	NA	NA	NA	NA
CRF	−0.116^e^	0.413^e^	NA	NA	NA	NA	NA	NA
2001								
Work ability^b^	0.089^f^	−0.041	0.078	NA	NA	NA	NA	NA
Absence^c^	−0.022	−0.006	−0.060^f^	−0.350^e^	NA	NA	NA	NA
2021								
WAI	−0.041	0.030	−0.004	−0.303^e^	−0.029	NA	NA	NA
LTPA	−0.019	0.106^f^	0.111^f^	−0.055	−0.012	0.135^e^	NA	NA
Strain^d^	0.065	0.002	0.015	−0.016	−0.094	−0.191^e^	−0.095^f^	NA
Education level	−0.025	0.039	−0.021	0.113	−0.003	0.175^e^	0.033	−0.269^e^

Abbreviations: BMI, body mass index; CRF, cardiorespiratory fitness; LTPA, leisure-time physical activity; MF, musculoskeletal fitness; NA, not applicable; WAI, work ability index.

^a^ Data are correlation coefficients. Correlations are biserial if work ability in 2001 is included, polyserial if strain in 2021 is included, and Pearson product moment for all others.

^b^ Coefficient is an inverted (multiplied by −1) version of the variable decreased work ability and overall functioning due to health impairment in 2001.

^c^ Refers to absenteeism due to sickness or injury.

^d^ Refers to work-related physical strain.

^e^ *P* < .01.

^f^ *P* < .05.

Baseline body mass index (BMI; calculated as weight in kilograms divided by height in meters squared), CRF, and MF *z* scores between dropouts and nondropouts were evaluated using the Little missing completely at random test. The test indicated that nondropouts had healthier characteristics than dropouts (eTable 4 in Supplement 1); that is, the missing values were not completely missing at random. Using weighted least squares mean and variance estimator in the path analysis, the missing completely at random assumption could be relaxed, and it can be assumed that the missing values were missing at random.

### Longitudinal Associations of Health-Related Fitness in Adolescence With Work Ability in Early and Late Middle Age

**Figure 2.**
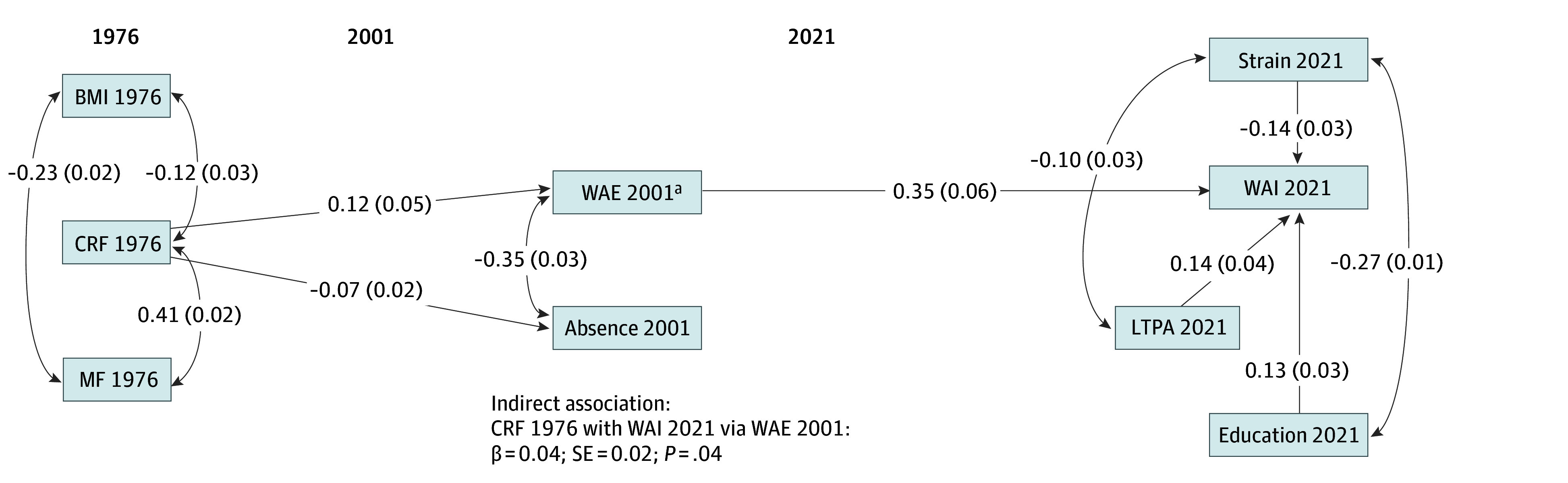
Path Diagram Diagram shows statistically significant (*P* < .05) standardized regression coefficients (straight arrow), correlations (curved arrow), and SEs (in parentheses). Absence 2001 refers to absenteeism due to sickness or injury in 2001. Strain 2021 refers to work-related physical strain in 2021. Education 2021 refers to education level in 2021. BMI indicates body mass index; CRF, cardiorespiratory fitness; LTPA, leisure-time physical activity; MF, musculoskeletal fitness; WAE, work ability estimate; WAI, Work Ability Index. ^a^Refers to an inverted (multiplied by −1) version of the variable decreased work ability and overall functioning due to health impairment in 2001.

The model fit information indicated that the path model fitted the data well (χ^2^_10_ = 8.84; *P* = .55; root mean square error of approximation = 0.00; comparative fit index = 1.00; Tucker-Lewis index = 1.00, standardized root mean square residuals = 0.02). The results (Figure 2 and eTable 1 in Supplement 1), adjusted for late middle age LTPA, educational level, and work-related physical strain, showed that CRF in adolescence was associated with the work ability indicators in both early and late middle age. Between adolescence and early middle age, adolescent CRF showed a direct positive association with work ability (839 participants; β = 0.12; SE = 0.05; 95% CI, 0.01 to 0.22; *P* = .03) and a negative association with sickness absence (834 participants; β = −0.07; SE = 0.02; 95% CI, −0.12 to −0.02; *P* = .004). However, although statistically significant, the associations were low in magnitude. Neither MF nor BMI was associated with early middle age work ability (MF, 1192 participants; β = −0.07; 95% CI, −0.17 to 0.03; BMI, 1207 participants, β = 0.09; 95% CI, −0.004 to 0.19) or sickness absence (MF, 1185 participants, β = 0.02; 95% CI, −0.03 to 0.06; BMI, 1202 participants, β = −0.03; 95% CI, −0.09 to 0.03) or late middle age work ability, mediated by work ability in early middle age (MF, 603 participants, β = −0.02; 95% CI, −0.06 to 0.01; BMI, 603 participants, β = 0.03; 95% CI, −0.004 to 0.07). Although no direct association between adolescent fitness and late middle age WAI was detected, a significant indirect association (603 participants; β = 0.04; SE = 0.02; 95% CI, 0.001 to 0.08; *P* = .04) was found between adolescent CRF and late middle age WAI mediated by full (ie, not impaired) work ability in early middle age. Analysis of the associations between early and late middle age showed that work ability in early middle age, which was concurrently a significant mediator in the indirect association between adolescent CRF and work ability in late middle age, had a medium-sized positive association (β = 0.35; SE = 0.06) with the WAI in late middle age.

## Discussion

This 45-year prospective cohort study contributes 4 major findings to the literature. First, higher adolescent fitness, particularly CRF, was directly associated with higher work ability and lower sickness absence in early middle age and was indirectly associated with higher WAI toward the end of working life (ages 57-64 years). Second, unlike previous studies that have investigated the associations of fitness with a disability pension and only among men, our study provides novel evidence on the prognostic value of CRF for future work ability in both men and women. Third, although small, the associations found were consistent and independent of potentially important confounders, such as BMI in adolescence and LTPA, educational level, and work-related physical strain in late middle age. Fourth, when CRF, MF, and BMI were all included in the model as exposure variables and therefore adjusted for each other, only CRF was associated with future work ability outcomes.

Our finding that CRF in adolescence was positively associated with the work ability indicators in early and late middle age is unique owing to its 45-year-long follow-up and responds to concerns raised in the public domain about young people’s decreased fitness^36^ in relation to their future health and work ability. We cannot directly compare our results with those of previous studies, because we found no similar studies with work ability as the primary outcome. However, our findings are supported by previous longitudinal studies^32,34,35^ with chronic disability as an outcome measure that have found an association between late adolescent CRF and an adulthood disability pension due to cardiovascular disease^34^ and all causes,^32^ independently of MF and BMI. In those studies, BMI was also a factor associated with a disability pension, whereas the role of MF remained smaller and more inconsistent.

The association of adolescent CRF with work ability in adulthood is likely due to the interrelationship between CRF and chronic cardiometabolic conditions^21,44,45,46^ that negatively influence work ability.^2,47^ It is widely accepted that in adulthood CRF is an important individual factor associated with cardiometabolic disease,^17,48^ which is a major burden for public health globally.^49,50^ Moreover, CRF in youth may contribute to the risk for metabolic syndrome,^21,51^ cardiovascular disease,^44,45^ and type 2 diabetes^52^ later in life. This pattern was clearly visible in our results, where adolescent CRF, mediated by work ability in early middle age, was positively associated with late middle age work ability. Although fitness status was not controlled for in middle age, the association of CRF with health conditions was present, as was that of health conditions with work ability. However, although the association of adolescent CRF with adulthood cardiometabolic health has been established,^21,26,44,45,51,52^ its mechanism remains unclear. It may be related to genetic regulation, which has been demonstrated not only to determine maximal oxygen uptake in youth^53^ but also likely contributes to a lower risk for cardiometabolic diseases in adulthood.^54^ In turn, Henriksson et al^32^ found an interesting association of youth CRF with not only disability due to circulatory causes but also tumors, injuries, and psychiatric, musculoskeletal, and nervous system disorders, indicating a possible broader role for CRF as a contributor to work ability. In addition to its genetic component, CRF is known to be associated with environmental factors, especially physical activity, meaning that higher CRF is also indicative of long-term exposure to an active life, which can also directly and indirectly positively influence health and, thus, work ability. Furthermore, better CRF is a marker of better brain (mental and cognitive) health,^55^ which is another important component of work ability.

We found no significant associations of adolescent MF or BMI with early or late middle age work ability after adjusting for CRF. Regarding MF, the result is mainly in line with previous findings. Despite Henriksson et al^33^ finding a significant association of late adolescent MF with all-cause disability, the association with disability pension due to cardiovascular disease was small and inconsistent.^34^ Similarly, the positive association of adulthood cardiometabolic health with youth MF^23^ has been smaller than that with CRF.^45,52,56,57,58^ Unlike previous corresponding cross-sectional^3,31^ and longitudinal^32,34^ studies, and studies on the role of youth body composition in future adulthood cardiometabolic disease risk,^22,25,44,45^ we observed no association of BMI with work ability after adjusting for CRF. Presumably, the conflicting finding might be related to methodological differences between our and the reference studies, as well as the relatively small sample size in our study. We assessed CRF by a running test, unlike the bicycle test used in the reference studies. Because running is a more weight-dependent activity than stationary biking, it might be that CRF and BMI were more interdependent in our study, a possibility that could partially explain the results. Moreover, only 2 of the reference studies controlled for CRF,^32,34^ indicating that further research on CRF adjustment is needed.

In addition to the WAI across 45 years, our path analysis yielded another major finding. Adolescent CRF, but not BMI or MF, was inversely and significantly associated with early middle age sickness absence. Because sickness absence, aside from being a main component of work ability,^41^ is a significant independent factor threatening productivity,^12,13^ this is an important finding that supplements the evidence gathered from studies with shorter follow-ups.^29,59^

The main strength of this prospective study is its 45-year-long follow-up, connecting pediatric age with age at the end of working life. The end-point age (57-64 years) is highly relevant from the work productivity viewpoint. This finding is an important addition to the literature^32,33,34,35^ on the longitudinal associations of fitness with chronic disability and early pensions. Along with economic impact, work ability is associated with quality of life.^5,6^ Hence, the results of this study highlight the role of physical activity in youth, a main lifestyle contributor to a better CRF, as an important factor to enhance well-being in adulthood. Other strengths are a sex-balanced geographically representative baseline sample that allowed us to investigate potential sex differences and similarities, and the use of sophisticated statistical techniques to address survivor bias, which is a common threat to validity in longitudinal studies. Moreover, the baseline fitness data were gathered using an objective measure, thereby enhancing their validity. Another strength is that work ability at the end point was measured with the widely used and validated WAI,^41,47,60^ which has been shown to be valid, especially among older workers,^2^ as was also the case in this study. Finally, measurements were conducted at 3 time points instead of just 2, allowing us to examine potential mediators in early middle age that influence work ability at the end of working life.

### Limitations

This study has its limitations. First, the WAI was not used in 2001, compelling us to resort to the simpler and discriminant measure of Work Ability Estimate in early middle age. However, despite the measure’s limitations, particularly in terms of lack of work-specific and prognostic perspective on work ability, it has been used widely^42,43^ and showed good concurrent validity with the WAI.^2^ Not surprisingly, the greatest association in our model was found between the Work Ability Estimate and the WAI. Second, the lack of fitness measurements in the follow-ups prevented us from investigating changes in CRF in relation to work ability, a task that remains for future studies. Third, the baseline measurement did not include in-depth health screening with assessment of all preexisting medical conditions (even though adolescents at these ages are mostly healthy) or family health history, which might have affected the associations between fitness and work ability. Fourth, the possible confounders were not controlled for in early middle age, which might have influenced the results concerning the first 25 years. Fifth, participant dropout, which is unavoidable in such a long follow-up, and the subsequent decline in sample representativeness should be noted. Moreover, the observational design means that causal relationships cannot be established.

## Conclusions

The results of this 45-year prospective cohort study provide novel findings that low CRF in adolescence is associated with poorer work ability at the end of working life, and, hence, with productivity and other economic factors. This association was independent of potentially relevant confounders, including BMI and MF, and was mediated by work ability in early middle age. An additional important contribution of this study is that we include both sexes, unlike earlier cohorts studying disability only in men, and our findings support that the long-term association between adolescent CRF and future work ability is consistent in male and female individuals. On the other hand, a significant association between BMI or MF with work ability was not observed later in life, after adjustment for CRF. Altogether, our findings support the notion that assessing CRF in adolescence is relevant and informative from a clinical, public health, and economic value. Furthermore, our findings suggest that promoting CRF through physical activity and exercise in childhood and adolescence could potentially benefit the work ability of the future labor force.

## Notes

Supplement 1. **eTable 1.** Associations in the Path Analysis, Adjusted for All Covariate **eTable 2.** Descriptions of the Fitness Tests in Adolescence **eTable 3.** The Work Ability Index, Items and Instructions for Calculation **eAppendix.** Sensitivity Analysis (Estimation of the Path Model Using Multigroup Model) **eTable 4.** Missing Value Analysis of Follow-Up and Non-Follow-Up Participants

Supplement 2.
Data Sharing Statement
