# Supplementary material for: Adolescent Cardiorespiratory Fitness and Future Work Ability
Source: JAMA Netw Open. 2024 Mar 27;7(3):e243861. doi: 10.1001/jamanetworkopen.2024.3861 (PMC10973892; doi:10.1001/jamanetworkopen.2024.3861)
Supplement: Supplement 1. — eTable 1. Associations in the Path Analysis, Adjusted for All Covariate eTable 2. Descriptions of the Fitness Tests in Adolescence eTable 3. The Work Ability Index, Items and Instructions for Calculation eAppendix. Sensitivity Analysis (Estimation of the Path Model Using Multigroup Model) eTable 4. Missing Value Analysis of Follow-Up and Non-Follow-Up Participants [file jamanetwopen-e243861-s001.pdf]

Supplemental Online Content

Laakso PTT, Ortega FB, Huotari P, Tolvanen AJ, Kujala UM, Jaakkola TT. Adolescent cardiorespiratory fitness and future work ability. *JAMA Netw Open*. 2024;7(3):e243861. doi:10.1001/jamanetworkopen.2024.3861

eTable 1. Associations in the Path Analysis, Adjusted for All Covariate
eTable 2. Descriptions of the Fitness Tests in Adolescence
eTable 3. The Work Ability Index, Items and Instructions for Calculation
eAppendix. Sensitivity Analysis (Estimation of the Path Model Using Multigroup Model)
eTable 4. Missing Value Analysis of Follow-Up and Non-Follow-Up Participants

This supplemental material has been provided by the authors to give readers additional information about their work.

© 2024 Laakso PTT et al. *JAMA Network Open.*

Exposure	Outcome	N	Standardized ß (95% CI)	P
**DIRECT ASSOCIATIONS**				
BMI 76	WA 01[a]	1207	.09 (<.00 to .19)	.06
MF 76	WA 01[a]	1192	-.07 (-.17 to .03)	.18
CRF 76	WA 01[a]	839	.12 (.01 to .22)	.03
BMI 76	ABSENCE 01	1202	-.03 (-.09 to .03)	.37
MF 76	ABSENCE 01	1185	.02 (-.03 to .06)	.47
CRF 76	ABSENCE 01	834	-.07 (-.12 to -.02)	.004
BMI 76	WAI 21	647	-.07 (-.14 to >.00)	.06
MF 76	WAI 21	644	.04 (-.06 to .14)	.40
CRF 76	WAI 21	404	-.07 (-.19 to .05)	.28
WA 01[a]	WAI 21	603	.35 (.23 to .47)	<.001
ABSENCE 01	WAI 21	603	.09 (-.01 to .20)	.09
LTPA 21	WAI 21	733	.14 (.07 to .21)	<.001
STRAIN 21	WAI 21	769	-.14 (-.21 to -.08)	<.001
EDUCATION 21	WAI 21	769	.13 (.08 to .18)	<.001
BMI 76	LTPA 21	743	.01 (-.08 to .10)	.79
MF 76	LTPA 21	743	.07 (-.02 to .16)	.14
CRF 76	LTPA 21	454	.09 (-.03 to .21)	.15
WA 01[a]	LTPA 21	692	-.07 (-.18 to .04)	.19
ABSENCE 01	LTPA 21	690	-.03 (-.14 to .08)	.57
INDIRECT ASSOCIATIONS				
BMI 76	WAI 21 via WA 01[a]	603	.03 (<.00 to .07)	.08
MF 76	WAI 21 via WA 01[a]	603	-.02 (-.06 to .01)	.20
CRF 76	WAI 21 via WA 01[a]	603	.04 (>.00 to .08)	.04
BMI 76	WAI 21 via ABSENCE 01	603	<.00 (-.01 to >.00)	.42
MF 76	WAI 21 via ABSENCE 01	603	>.00 (<.00 to .01)	.48
CRF 76	WAI 21 via ABSENCE 01	603	-.01 (-.02 to >.00)	.15
BMI 76	WAI 21 via LTPA 21	733	>.00 (-.01 to .01)	.79
MF 76	WAI 21 via LTPA 21	733	.01 (<.00 to .02)	.16
CRF 76	WAI 21 via LTPA 21	454	.01 (-.01 to .03)	.21

eTable 1. Associations in the Path Analysis, Adjusted for All Covariates

© 2024 Laakso PTT et al. *JAMA Network Open.*

Abbreviations: BMI 76, body mass index in 1976; MF 76, musculoskeletal fitness in 1976; CRF 76, cardiorespiratory fitness in 1976; WA 01, work ability in 2001; ABSENCE 01, absenteeism due to sickness or injury in 2001; WAI 21, work ability index in 2021; LTPA 21, leisure-time physical activity in 2021; STRAIN 21, work-related physical strain in 2021; EDUCATION 21, education level in 2021

[a]An inverted (multiplied by -1) version of the variable "Decreased work ability and overall functioning due to health impairment in 2001"

© 2024 Laakso PTT et al. *JAMA Network Open.*

Test	Measured fitness component	Method	Scoring	Environment
**Musculoskeletal fitness:**				
Sit-ups	Abdominal and hip flexor muscular endurance	Rise from lying on back, knees bent at 90 degrees, both feet on floor held down by a partner, fingers interlocked behind the head, until the upper body is vertical and then lowered until back touches the floor. Do as many repetitions as possible in 30 seconds.	Total number of repetitions in 30 seconds	Indoor sport facility
Standing broad jump	Explosive leg power	Single two-foot jump with legs in parallel position in take-off and landing.	Distance in centimeters	Indoor sport facility
Flexed arm hang (girls)	Upper body strength and endurance	Hang with underhand grip as long as possible in a stable position with chin above the horizontal overhead bar.	Time in seconds	Indoor sport facility
Pull-ups (boys)	Upper body strength	Grasp the horizontal overhead bar with overhand grip, arms fully extended. Raise the body by flexing arms until chin tops the bar. Lower body again until arms are fully extended. Repeat as many times as possible.	Total number of repetitions	Indoor sport facility
**Cardiorespiratory fitness:**				
1 500 meters running (girls)	Cardiorespiratory fitness	1 500 m running in shortest possible time	Time in seconds	Outdoor 400 m running track
2 000 meters running (boys)	Cardiorespiratory fitness	2 000 m running in shortest possible time	Time in seconds	Outdoor 400 m running track

eTable 2. Descriptions of the Fitness Tests in Adolescence

© 2024 Laakso PTT et al. *JAMA Network Open.*

Item	Points
Current work ability compared with the lifetime best	0-10
Work ability in relation to the demands of the job	2-10
Number of current diseases diagnosed by a physician	1-7
Estimated work impairment due to diseases	1-6
Sick leave during the past year	1-5
Own prognosis of work ability two years from now	1, 4 or 7
Mental resources	1-4

eTable 3. The Work Ability Index, Items and Instructions for Calculation (Ilmarinen 2007, van den Berg et al. 2009).
The index is calculated by summing the item points (range: 7-49) and can be divided into the categories of poor (7–27 pts), moderate (28–36 pts), good (37–43 pts), and excellent (44–49 pts) work ability.

© 2024 Laakso PTT et al. *JAMA Network Open.*

eAppendix. Sensitivity Analysis (Estimation of the Path Model Using Multigroup Model)

Estimation of the path model using multi group model

According to research question in which we wanted to test whether there is age or sex differences in the associations between fitness and work ability, we first estimated a multigroup model for two age groups (12-15 and 16-19-years-olds in baseline) in both sexes. The model fit information [$\chi^2(73)$=98.07, p=.03; RMSEA=0.02; CFI=0.96; TLI=0.91, SRMR=0.07] shows that the model fitted well for the data. To detect the possible differences between the groups, all path coefficients were set equal between age and sex groups and modification indices used to reveal the differences (index value > 10 indicates possible difference).

Three modification indices [males 12-15y (association: CRF76-ABSENCE01) MI=10.13; females 12-15y (associations: CRF76-STRAIN21, MF76-ABSENCE01) MI=11.96, MI=10.25) were larger than ten. The estimates for 12-15-year-old males were -.11 and -.15 for standardized and constrained coefficients, respectively, and for 12-15-year-old females .19 and .09. The results indicate no significant difference between using multi group and single group model. Due to higher statistical power and good model fit (=8.84, p=.55; RMSEA=0.00; CFI=1.00; TLI=1.00, SRMR=0.02), the single group model was adopted as the final model.

© 2024 Laakso PTT et al. *JAMA Network Open.*

		BMI76	CRF76	MF76
**Work ability 01**	Follow-ups	19.7	.06	.15
	non-follow-ups	20.0	-.12	-.29
	P	.054	.004[b]	<.001[a]
**Absence 01**	Follow-ups	19.7	.06	.16
	non-follow-ups	20.0	-.11	-.30
	P	.023[b]	.007[b]	<.001[a]
**Work ability index 21**	Follow-ups	19.6	<.00	.05
	non-follow-ups	19.9	<.00	-.02
	P	.033[b]	.918	.514

eTable 4. Missing Value Analysis of Follow-Up and Non-Follow-Up Participants
Values = means for BMI76; age & sex standardized z-score means for CRF76 and MF76.
[a]p<.01
[b]p<.05
Little's MCAR test $[\chi^2(240) = 427.58, p < .001]$ indicated that missing values were not completely missing at random (MCAR). Using WLSMV estimator in the path analysis, the MCAR assumption can be relaxed, and it can be assumed that the missing values were missing at random (MAR)

© 2024 Laakso PTT et al. *JAMA Network Open.*
