# Supplementary material for: Adolescent Cardiorespiratory Fitness and Future Work Ability
Source: JAMA Netw Open. 2024 Mar 27;7(3):e243861. doi: 10.1001/jamanetworkopen.2024.3861 (PMC10973892; doi:10.1001/jamanetworkopen.2024.3861)
Supplement: Supplement 2. — Data Sharing Statement [file jamanetwopen-e243861-s002.pdf]

# Data Sharing Statement

Laakso. Adolescent Cardiorespiratory Fitness and Future Work Ability. *JAMA Netw Open*. Published March 27, 2024. doi:10.1001/jamanetworkopen.2024.3861

**Data**
**Data available:** No

**Additional Information**
**Explanation for why data not available:** Research data includes sensitive information
